# Supplementary material for: Operationalising effective coverage measurement of facility based childbirth in Gombe State; a comparison of data sources
Source: PLOS Glob Public Health. 2022 Apr 21;2(4):e0000359. doi: 10.1371/journal.pgph.0000359 (PMC10021305; doi:10.1371/journal.pgph.0000359)
Supplement: S1 Table — (DOCX) [file pgph.0000359.s001.docx]

# S1 Table: Components used to define each step of the coverage cascade for the two different data sources

| **Step of cascade** | **Measure** | **(1) NDHS and project data** | **(2) NDHS and routine data** |
| --- | --- | --- | --- |
| Target population (population in need of the intervention) | Women who report a live birth | NDHS: live birth in the last 5 years | NDHS: live birth in the last 5 years |
| Service contact coverage (attends a health facility for birth) | Women report where they gave birth | NDHS: place of delivery   - Home (respondent’s or other) - Govt. health centre/post - Govt. hospital/private hospital or clinic | NDHS: place of delivery   - Home (respondent’s or other) - Govt. health centre/post - Govt. hospital/private hospital or clinic |
| Input adjusted coverage (health facility ready to deliver care):   1. Facility infrastructure | Communication | HF survey: Any means of communicating with another facility   - Facility landline/mobile phone - Staff member mobile phone; - Phone outside the facility; OR - Radio. |  |
|  | Light source | HF survey: Functioning electricity supply on the day of the survey (either an electricity connection or an alternative power supply) OR a 24-hour functioning light source available in the labour ward. |  |
|  | Sanitation | HF survey: Toilets accessible to female facility users |  |
|  | Water supply | HF survey: Source of clean running water (e.g. bucket and plug or piped water) |  |
| 1. Staffing & training | Skilled birth attendance | HF survey: Last seven days facility had at least one midwife/clinician available 24 hours a day, 7 days a week | DHIS2: All deliveries attended by a skilled birth attendant |
| 1. Drugs & commodities | Anticonvulsants | HF survey: Magnesium sulphate available in the facility on the day of the survey | DHIS2: no stock out of magnesium sulphate in the past one month |
|  | Blood pressure machine | HF survey: Blood pressure machine (sphygmomanometer) available in the service area. |  |
|  | Delivery pack | HF survey: Clamp or umbilical tie, sterile scissors or blade; AND suture material with needles available in the service area. |  |
|  | Newborn resuscitation device | HF survey: Functioning bag & mask size 0 (for preterms) AND size 1 (for term babies) available in the service area | DHIS2: no stock out of resuscitation equipment in the past one month |
|  | Gloves | HF survey: Disposable gloves available in the service area. |  |
|  | Infection control | HF survey: soap and water for the purposes of hand washing OR alcohol based hand rub inside the labour room. |  |
|  | Intravenous fluids and infusion set | HF survey: Intravenous fluids with infusion set available in the service area |  |
|  | Scale | HF survey: Accessible and working baby scale available in the service area. |  |
|  | Suction apparatus | HF survey: Newborn suction device OR mucus trap/suction machine available in the service area |  |
|  | Uterotonic | HF survey: oxytocin, misoprostol OR ergometrine available in the facility on the day of the survey | DHIS2: no stock out of uterotonic (either oxytocin or misoprostol) in the past one month |
| Intervention coverage (receives services and medication) | Administers uterotonic | CO: Birth attendant observed to administer uterotonic | NDHS: Woman reports received injection immediately after delivery  ***DHIS2: Active Management of 3^rd^ stage of labour*** |
|  | Baby weighed | CO: Baby’s weight observed to be recorded | NDHS: Weighed at birth based on health card or mother’s recall  ***DHIS2: Birth weight recorded for all live births*** |
|  | Thermal care | CO: Birth attendant observed to dry baby immediately with towel, place the newborn on mother’s abdomen or if not placed skin-to-skin to wrap baby in dry towel AND baby not bathed within the first hour after birth | NDHS: Women reports child put on mother's chest and bare skin after birth AND baby not bathed within the first hour of birth.  ***NDHS: baby wiped dry in as few minutes after birth***  ***DHIS2: Babies put to breast within 1hr with skin-to-skin to keep warm*** |
| Quality-adjusted coverage (receives timely, appropriate, responsive and respectful care) | Monitoring progress | CO: Birth attendant observed to take maternal blood pressure |  |
|  | Explain what will happen in labour | CO: Birth attendant observed to explain procedures to woman (support person) before proceeding |  |
|  | Support person present | CO: A support person (companion) for mother is observed to be present at birth |  |
|  | Patient satisfaction with care received | CO: Woman would recommend someone else to deliver in the health facility |  |

TABLE NOTE: HF=health facility assessment, CO=clinical observations. Items highlighted in **bold and italicised** were not available: DHIS2 these indicators are captured at facility level but not included in monthly monitoring reports and NDHS data on immediate drying has not been made available in the publically available recode dataset.
