# Supplementary material for: Operationalising effective coverage measurement of facility based childbirth in Gombe State; a comparison of data sources
Source: PLOS Glob Public Health. 2022 Apr 21;2(4):e0000359. doi: 10.1371/journal.pgph.0000359 (PMC10021305; doi:10.1371/journal.pgph.0000359)
Supplement: Attachment — Submitted filename: Response to Reviewers.docx [file pgph.0000359.s003.docx]

Dear Editor,

We would like to extend our thanks to the reviewers for their time to review our manuscript. In response we have made the following key changes:

- We have updated the title to “*“Measuring effective coverage of facility based childbirth in Gombe State; a comparison of data sources”*
- We have clarified out data availability statement to make it clear that the data is freely available and to provide the link to LSHTM’s in-house open access research repository “Data Compass” to guide the reader to where it can be accessed.
- We have reviewed and updated the reference list
- We have carefully reviewed the manuscript to ensure consistency in the use of past tense and proof read the manuscript.

We provide detailed response to the editorial and reviewer comments point by point in the table below.

Yours sincerely

Josephine Exley, on behalf of the study authors

| Journal Requirements: |  |
| --- | --- |
| 1. Please include a complete copy of PLOS’ questionnaire on inclusivity in global research in your revised manuscript. Our policy for research in this area aims to improve transparency in the reporting of research performed outside of researchers’ own country or community. The policy applies to researchers who have travelled to a different country to conduct research, research with Indigenous populations or their lands, and research on cultural artefacts. The questionnaire can also be requested at the journal’s discretion for any other submissions, even if these conditions are not met.  Please find more information on the policy and a link to download a blank copy of the questionnaire here: [https://journals.plos.org/plosone/s/best-practices-in-research-reporting](https://protect-eu.mimecast.com/s/ugsfCO8KBCvoPVDiEg4zi?domain=journals.plos.org). Please upload a completed version of your questionnaire as Supporting Information when you resubmit your manuscript. | We have now completed this form and have included in our resubmission. |
| 2. Please update your Competing Interests statement. If you have no competing interests to declare, please state: “The authors have declared that no competing interests exist.” | The Competing Interests statement has now been revised as requested. |
| 3. In the online submission form, you indicated that “The data are available from the principal investigator of the IDEAS project and co-author for this manuscript, Dr Tanya Marchant ORCID id 0000-0002-4228-4334. Reuse permitted on request.”.  All PLOS journals now require all data underlying the findings described in their manuscript to be freely available to other researchers, either:   1. In a public repository, 2. Within the manuscript itself, or 3. Uploaded as supplementary information.   This policy applies to all data except where public deposition would breach compliance with the protocol approved by your research ethics board. If your data cannot be made publicly available for ethical or legal reasons (e.g., public availability would compromise patient privacy), please explain your reasons by return email and your exemption request will be escalated to the editor for approval. Your exemption request will be handled independently and will not hold up the peer review process, but will need to be resolved should your manuscript be accepted for publication. One of the Editorial team will then be in touch if there are any issues. | All data associated with the analysis are stored on the LSHTM in-house open access research repository “Data Compass”. The data is freely available but users are required to complete a request form before access is granted.  We have now updated the wording in the manuscript to make it clearer that the data is freely available and to direct potential users to where they can access the data:  “*The comprehensive health facility project data is freely available through LSHTM Data Compass. It can be accessed online:*  <https://doi.org/10.17037/DATA.00001712> |
| 4. Please review your reference list to ensure that it is complete and correct. If you have cited papers that have been retracted, please include the rationale for doing so in the manuscript text, or remove these references and replace them with relevant current references. Any changes to the reference list should be mentioned in the rebuttal letter that accompanies your revised manuscript. If you need to cite a retracted article, indicate the article’s retracted status in the References list and also include a citation and full reference for the retraction notice. | No references have been retracted.  At the time of submission, reference was made to two submitted papers which have now been published; references have been updated accordingly:  15. Exley J, Gupta PA, Schellenberg J, Strong K, Requejo J, Moller AB, et al. A rapid systematic review and evidence synthesis of effective coverage measures and cascades for childbirth, newborn and child health in low- and middle-income countries. Journal of global health. 2021;12  24. Willey B, Umar N, Beaumont E, Allen E, Anyanti J, Bello AB, et al. Improving maternal and newborn health services in Northeast Nigeria through a government-led partnership of stakeholders: a quasi-experimental study. BMJ Open. 2022;12(2):e048877 |
| Reviewer #1: |  |
| To the best of what has been presented; the methods and materials used are appropriate to the research objectives. The data analysis techniques used are vigorous and the conclusions have been based on the findings. Therefore, the article meets APLOS publication criteria and is very informative for health practice. | We thank the reviewer for their comments |
| Reviewer #2 |  |
| (a) Title: The title is not clear. Whereas the study reports findings related to optimising metrics of coverage of maternal health services, this is not accurately represented in the title. I suggest that you include the word(s) "indicator", "metric", "measurement" or whichever you may deem appropriate to clarify that the manuscript content regards metrics and not implementation per se. The term "operationalising" in the title contributes to ambiguity. | We have added measurement, so the title now reads: *“Operationalising effective coverage measurement of facility based childbirth in Gombe State; a comparison of data sources”* |
| (b) Abstract: Whereas the abstract is concise, you need to revise to ensure consistence of tense. Since this is not a study protocol, I suggest using past tense consistently through the manuscript. | We have reviewed and edited the abstract and manuscript to ensure that we have consistently used the past tense. |
| (c) Introduction: Lines 54-57. I suggest that you limit this section to clearly stating the study aims. The study design descriptions, such as, ".... using comprehensive facility assessment project data....", can be detailed in the methods section. | We have reviewed the aims statement and made edits to reduce the description of the data sources used. |
| (d) Conclusions: Please revise this section to reflect concordance with study aims. Whereas it is true that effective coverage of childbirth care is low in Gombe, this had already been demonstrated from the NDHS, albeit sub-optimally. What then should the reader draw from your work? How did the triangulation of data sources improve (potential) utility of routinely available data? | The NDHS does not report effective coverage of childbirth care that moves beyond contact coverage by adjusting the proportion of women who delivered at a health facility for some measure of the quality of care. Here we define effective coverage as a care cascade comprising of 6 steps as recommended by the WHO. To measure all steps of the cascade we need to combine population data with health facility data.  This study aimed to examine the feasibility of constructing effective coverage cascades of childbirth care using data typically available to decision makers. To do this we compared effective coverage measures constructed using comprehensive project data and routine data (DHIS2).  In the conclusions we address this aim. We highlight that both approaches (using project data or routine data) demonstrate that effective coverage is low in this setting, driven by low attendance at a facility for childbirth and poor availability of the essential inputs.  However, we found that it was only feasible to partially construct effective coverage using routine data as no measures of process of care are currently captured in routine data. If we want to increase the uptake of effective coverage measures, then there is a need to advocate for the inclusion of these measures in routine data sources to optimise these data sources. |
| (e) Study data: please make the study data available or otherwise state justification for not doing so. | See comment above. We have revised statement to make it clear that the data is publically available. |
| Reviewer #3: This study used a combination of facility data to estimate the effective coverage of the childbirth care system in Gombe State, Nigeria. The study found effective coverage estimates for different data combinations and discussed the strength and weaknesses of their method. This adds to the current body of evidence in Gombe State as regards the effective coverage for maternal, newborn, child, and adolescent health and nutrition (MNCAHN). The result is well presented and discussed. | We thank the reviewer for their comments. |
| (1.) The authors should consider discussing more on how they managed to adjust for the sample design for multiple data, given that the design could be different for different survey data. | We have followed established methods for calculating the CI around linked data, see:  Sauer, S. M., et al. (2020). "Variance estimation for effective coverage measures: A simulation study." J Glob Health **10**(1): 010506.  This methods paper is referenced in our manuscript should readers which to see further detail. |
| (2.)The authors should explicitly write out the governing formulas/ equations to calculate these measures, and if possible, share the code used in the computation for readers who wish to reproduce the result. | Individual step of the cascade (j), coverage (C) were defined as the proportion of the target population (M) for whom the condition of that particular stage was met (N):  $Cj=\frac{Nj}{Mj}$  To calculate coverage as progress along the cascade we multiplied the prevalence of the step by the prevalence of the previous step as recommended by previous studies. As the effective coverage estimation has become more prominent in recent years there are a number of articles describing and applying the method. See for example: Willey B, Waiswa P, Kajjo D, Munos M, Akuze J, Allen E, et al. Linking data sources for measurement of effective coverage in maternal and newborn health: what do we learn from individual- vs ecological-linking methods? Journal of global health. 2018;8(1):010601  We do not think that the inclusion of the formula substantially adds to the understanding of the analysis, rather it may make the analysis appear more complicated than is necessary.  The coding for the manuscript is dependent on the datasets in use rather than generic. We are happy to share the code upon request. |
| (3.) The authors should proofread again for minor grammatical errors. | We have carefully read through the article. Changes are shown in the tracked version of our resubmission. |
